# Macular morphological prognostic factors of myopic foveoschisis with foveal detachment

**DOI:** 10.3389/fmed.2025.1622151

**Published:** 2025-09-10

**Authors:** Mengai Wu, Lifeng Chen, Yuanyuan Fan, Li Lin, Zhijie Wang, Guanshun Yu, Xinyue Wu, Fan Lu, Bin Zheng

**Affiliations:** National Clinical Research Center for Ocular Diseases, Eye Hospital, Wenzhou Medical University, Wenzhou, China

**Keywords:** myopic foveoschisis, OCT, OCTA, en-face, microperimetry

## Abstract

**Purpose:**

To evaluate the optical coherence tomography (OCT) and optical coherence tomography angiography (OCTA) parameters as a prognostic indicator of visual outcome in patients with myopic foveoschisis (MF) and foveal detachment (FD).

**Methods:**

Twenty-three individuals with MF and FD and undergoing vitrectomy participated in this study. Preoperative OCT parameters were obtained, including central foveal thickness (CFT), foveal detachment thickness (FDT), foveoschisis thickness (FST), and subfoveal choroidal thickness (SCT). Preoperative optical coherence tomography angiography (OCTA) images were assessed to measure macular vascular parameters in both superficial capillary plexus (SCP) and deep capillary plexus (DCP). En-face OCTA images was also employed to determine the area and perimeter of the ellipsoid zone (EZ) fracture. Microperimetry was performed to evaluate macular sensitivity (MS) covering the central 20°, 10°, 2° field centered at fovea.

**Results:**

The best corrected visual acuity (BCVA) improved significantly six months after surgery. When comparing groups based on postoperative BCVA, those with BCVA ≥0.5 had a substantially lower FD height and a higher vessel density (VD) in the SCP than those with BCVA <0.5. Preoperative BCVA demonstrated significant correlations with CFT, the EZ disruption area, and the EZ disruption perimeter. Additionally, preoperative 20° MS, 10° MS, and 2° MS were separately correlated with SCT, whole DCP VD, parafoveal VD, as well as the EZ disruption area and perimeter. Multiple linear regression analysis revealed that postoperative BCVA at six months was significantly associated with preoperative BCVA and preoperative whole SCP VD.

**Conclusion:**

Our study highlights that in addition to preoperative visual acuity, OCTA may be a useful additional prognostic factor for predicting visual outcomes in patients with MF and FD after vitrectomy.

## Introduction

High myopia, defined as a refractive error of at least −6.00 diopters or an axial length of 26.5 mm or more, is an increasing public health concern worldwide. It is estimated that pathological myopia affects 0.9–3.1% of the global population. Myopic foveoschisis (MF) is a complicated and progressive disorder observed in highly myopic eyes, characterized by the separation of retinal layers, primarily in the macular region. MF tends to progress slowly and may remain stable for several years during its early stages. However, MF can progress to foveal detachment (FD) in 34.5–72% of patients (1–3), resulting in significant vision impairment (4). Vitrectomy is the primary treatment approach for patients diagnosed with MF (5). However, there is no standardized clinical protocol exists for managing MF (6). Current researches mainly concentrates on the surgical techniques that involve either the preservation or removal of the internal limiting membrane (ILM) (7, 8), the benefits of fovea-sparing ILM peeling (9, 10), and the necessity and effectiveness of gas tamponade during these procedures (11). However, there is limited research on the relationship between structure and visual function in patients with MF and FD. Currently, no specific structural parameters have been identified to effectively predict the visual outcomes for these patients.

Optical coherence tomography (OCT) is a non-invasive imaging technology that employs light waves to capture high-resolution, cross-sectional images of the retina. It has proven to be an invaluable tool in ophthalmic diagnostics and in monitoring postoperative recovery in patients undergoing various eye surgeries (12, 13). Several studies have identified an association between postoperative EZ disruption and visual outcomes in MF patients (14–16). Nonetheless, evaluating the extent of preoperative EZ disruption in patients with MF and FD was reported to be challenging (16, 17).

Optical coherence tomography angiography (OCTA) is a newly established three-dimensional OCT technique used for visualizing and assessing retinal blood vessels (18). The fluctuations of blood flow within the vessels are converted into decorrelated signals that can be distinguished from static tissue, enabling quantification of blood flow in the retina and choroid. Our previous research found that the retinal deep capillary plexus (DCP) vessel density (VD) in patients with MF and FD is significantly correlated with both 10° and 20° macular sensitivity (MS). Nonetheless, it is unclear if OCTA parameters can serve as reliable predictors of visual outcome after vitrectomy.

Therefore, the purpose of this study is to investigate the specific macular morphological characteristics of OCT, OCTA, and en-face OCT to predict postoperative visual acuity in patients with MF and FD.

## Materials and methods

### Participants

This study is a retrospective, consecutive study involving patients with MF and FD, who underwent PPV surgery at the Eye Hospital of Wenzhou Medical University from October 2019 to December 2023. The study follows the principles of the Declaration of Helsinki and was approved by the Institutional Review Board. Data were collected from hospital records, with no patient participation required. We analyzed 23 eyes from 23 patients with MF and FD who consecutively received PPV treatment at the Eye Hospital of Wenzhou Medical University. The inclusion criteria included: (1) MF with FD without macular hole: intraretinal schisis into a thicker inner layer and a thinner outer layer at the macula, along with an intraretinal column, which could be clearly detected on SD-OCT; (2) age over 18 years; (3) eyes with spherical equivalent (SE) ≤ −6.0 diopters (D) or axial length (AL) ≥ 26.5 mm; and (4) postoperative follow-up of over 6 months. The exclusion criteria included: (1) pre-existing retinal disease or macular pathology other than MF, such as diabetic retinopathy, choroidal neovascularization, uveitis, full-thickness macular hole; (2) any previous ophthalmic surgery besides cataract surgery; (3) insufficient resolution of OCT or OCTA scans. The following data were collected from medical records: age, gender, preoperative lens status, refractive error, axial length, preoperative best corrected visual acuity (BCVA), macular sensitivity (MS), preoperative OCT, OCTA, and postoperative OCT data.

### OCT image analysis

**Figure 1:**
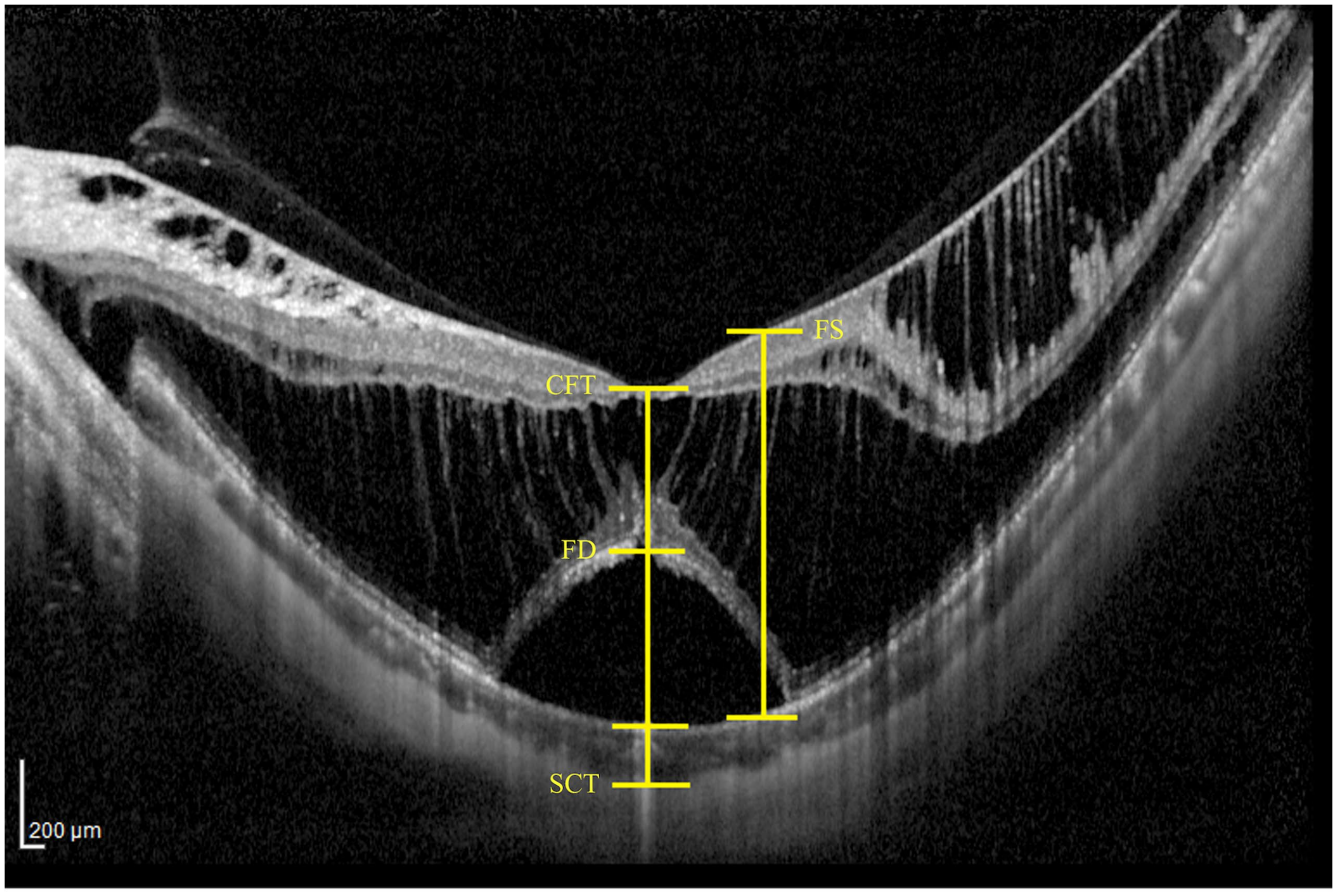
Representative examples of measuring OCT parameters, including central foveal thickness (CFT), subfoveal choroidal thickness (SCT), as well as the heights of foveal detachment (FDT) and foveoschisis (FST).

Preoperative horizontal line scan images centered at the fovea were acquired using spectral-domain optical coherence tomography (SD-OCT; Heidelberg Spectralis OCT, Heidelberg, Germany). These images were analyzed to evaluate key parameters, including central foveal thickness (CFT), subfoveal choroidal thickness (SCT), and the heights of foveoschisis (FST) and foveal detachment (FDT) (Figure 1). CFT was measured manually from the retinal pigment epithelium (RPE) to the inner surface of the retina at the fovea. SCT was defined as the distance between the base of the RPE and the choroidoscleral boundary at fovea. The height of FS was defined as the distance between the inner and outer boundaries of the neuroretina 1,000 μm from fovea. The height of FD was determined by measuring the maximum distance between the inner boundary of the RPE and the outer boundary of the neuroretina in the foveal region.

### OCTA image quantification

**Figure 2:**
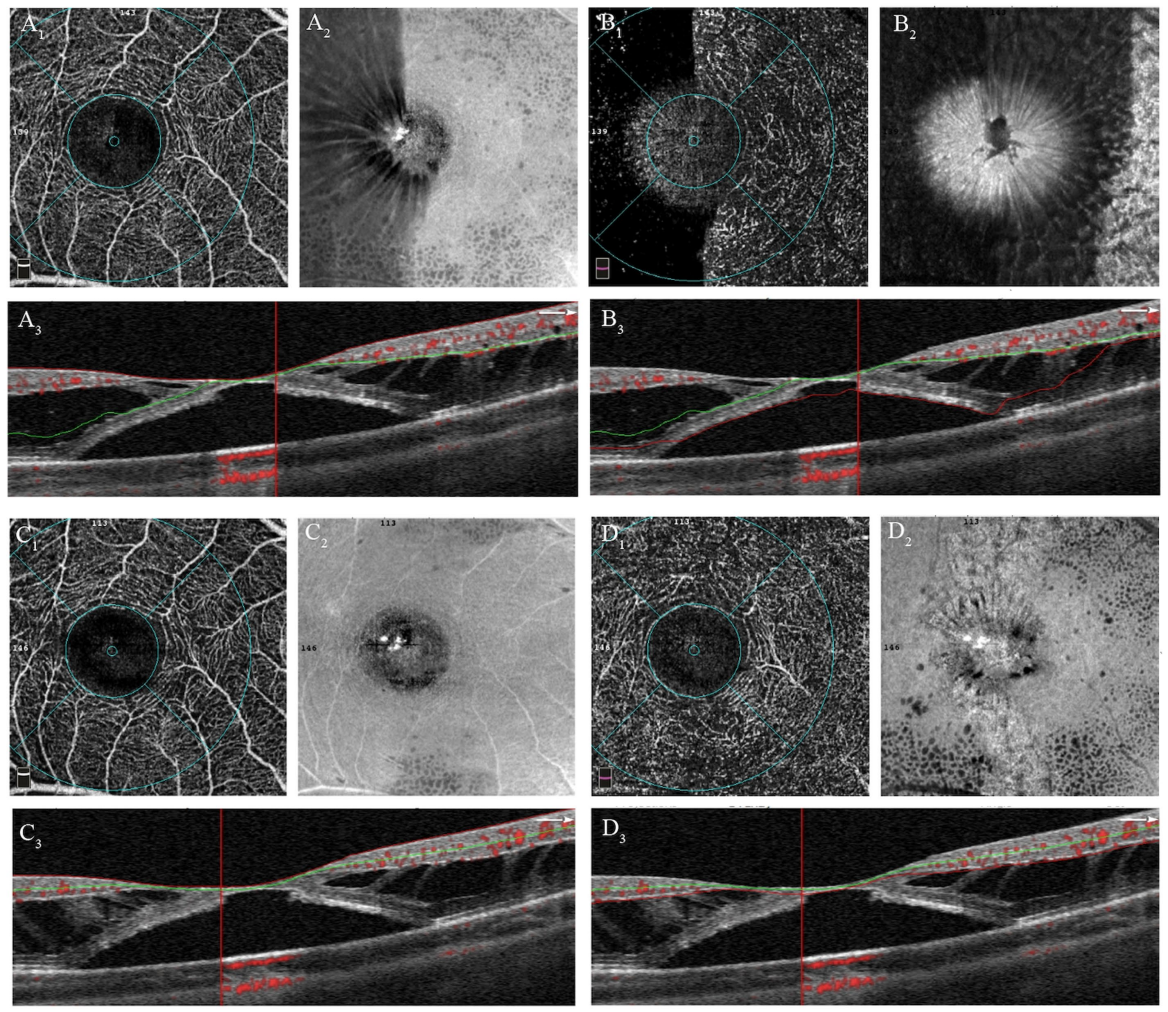
Manual adjustments of segmentation errors in OCTA images of patient with MF and FD. **(A**_**1**_**)** OCTA image of the SCP before adjustment; **(A**_**2**_**)** En-face image of SCP before adjustment; **(A**_**3**_**)** Correspondent B-scan image with segmentation lines of SCP, showing obvious segmentation errors. **(B**_**1**_**)** OCTA image of the DCP before adjustment; **(B**_**2**_**)** En-face image of DCP before adjustment; **(B**_**3**_**)** Correspondent B-scan image with segmentation lines of DCP, showing obvious segmentation errors. **(C**_**1**_**)** OCTA image of the SCP after adjustment; **(C**_**2**_**)** En-face image of SCP after adjustment; **(C**_**3**_**)** Correspondent B-scan image with correct segmentation lines of SCP. **(D**_**1**_**)** OCTA image of the DCP before adjustment; **(D**_**2**_**)** En-face image of DCP before adjustment; **(D**_**3**_**)** Correspondent B-scan image with correct segmentation lines of DCP.

The OCTA images with scan mode of 3 mm × 3 mm centered at fovea were obtained using an AngioVue Imaging System (RTVue XR Avanti, Optovue Inc., California, USA) with the 3D projection artifact removal approach. The retinal superficial capillary plexus (SCP) and deep capillary plexus (DCP) were automatically segmented by the built-in OCTA software. The SCP was defined with an inner boundary of the internal limiting membrane (ILM) and an outer boundary 10 μm above the inner plexiform layer (IPL) and inner nuclear layer (INL) junction. The DCP was segmented between the inner boundary 10 μm above the IPL and INL junction and the outer boundary 10 μm below the outer plexiform layer (OPL) and outer nuclear layer (ONL) junction. Due to separations in the retinal interlayer structure in patients with macular traction maculopathy (MTM), segmentation errors in OCTA images were common. Each examination was carefully reviewed and adjusted for segmentation lines at the ILM, IPL, and OPL, as shown in Figure 2. The vascular density (VD) parameters for the SCP and DCP were automatically determined by the AngioVue Analytics program for three different regions: the entire 3 mm x 3 mm area (whole VD), the foveal 1 mm x 1 mm area (foveal VD), and the annular zone ranging from 1 mm x 1 mm to 3 mm x 3 mm (parafoveal VD). The capillary-free area within the macula is known as foveal avascular zone (FAZ). The FAZ circularity index was calculated by dividing the actual perimeter of the FAZ by the perimeter of a standard circle with an equivalent area. Foveal density 300 (FD 300) refers to the VD measured from ILM to the OPL within a 300 μm region surrounding the FAZ. All values were computed automatically using the software.

### En-face image quantification

**Figure 3:**
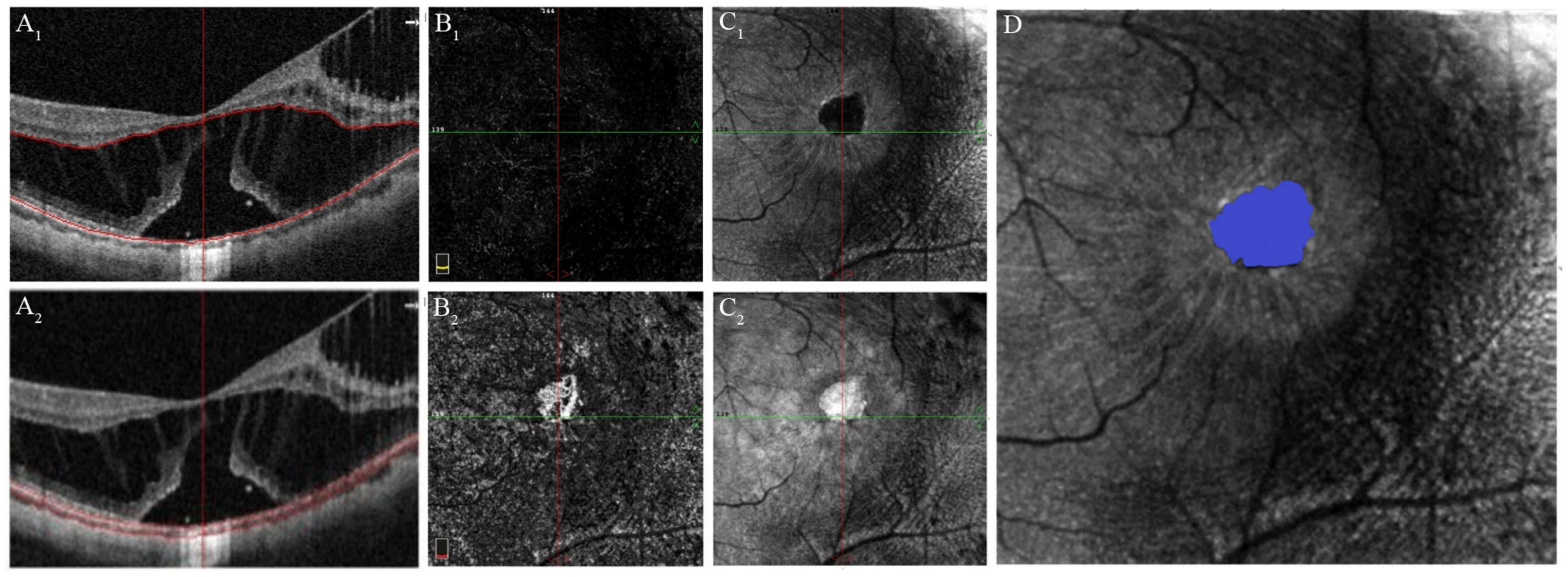
Quantification of EZ disruption through en-face imaging of the outer retina derived from the OCTA scan. **(A**_**1**_**)** OCT image with segmentation lines of outer retina. **(B**_**1**_**)** OCTA image of the outer retina shows absence of flow signal, similar to that observed in healthy individuals. **(C**_**1**_**)** En-face image of the outer retina, the EZ disruption area appears dark due to the lack of reflection in regions where the EZ band is missing. **(A**_**2**_**)** OCT image with segmentation lines of the choroid. **(B**_**2**_**)** OCTA image of the choroid shows increased flow signals in the region of the EZ defect. **(C**_**2**_**)** En-face image of the choroid shows that the EZ disruption area appears bright due to the “penetration effect” where the EZ band is missing. **(D)** Quantification of the area and perimeter of EZ disruptionm using the en-face image of the outer retina.

En-face images of the outer retina were automatically extracted from the OCTA scan using AngioVue software, delineating the inner boundary 10 μm below the junction of the OPL and ONL, while the outer boundary defined by the Bruch membrane (Figure 3). In the en-face outer retina images, the ellipsoid zone (EZ) disruption appears dark because of the absence of reflection where the EZ band is absent (19). En-face images of the choroid were also segmented from the Bruch membrane to 10 μm beneath it. In the en-face outer retina images, disruptions in the EZ appear light because of the penetration effect in the absence of the EZ band. However, we observed that the boundaries of EZ disruption region in the en-face images of the choroid were less distinct compared to those in the outer retina. Consequently, an independent qualified technician manually outlined the EZ disruption area in the outer retina using Image-Pro Plus 6.0 software (Media Cybernetics, Rockville, Maryland, USA). The software then automatically calculated the area and perimeter of the EZ disruption.

### Microperimetry acquisition

The MP-3 microperimeter (Nidek, Gamagori, Japan), a sophisticated microperimeter with a fundus image-tracking system, was used to assess retinal sensitivity. Microperimeter was performed in a dark room with pupil dilation and patching of the contralateral eye. The dull white background was set at a luminance of 10 cd/m^2^ and the maximum luminance of MP-3 was 3,183 cd/m^2^, providing a dynamic range of 0 to 34 decibels (dB). The stimulation pattern for the eye examination consisted of 61 stimulus points covering the central 20° field, with one point at the central fovea, four points at rings of 0.5°radii, eight points at rings of 1°, 2 °, 3 °radii, and sixteen points at rings of 4°, 5 °radii. The retinal sensitivity is expressed in dB.

### Surgical technique

PPV was carried out by a single surgeon (B.Z.) utilizing a 23G or 25G vitrectomy system (Alcon Constellation Vision System). For patients with significant cataracts, phacoemulsification with intraocular lens implantation was conducted. After core vitrectomy, if a posterior vitreous detachment had not yet occurred, it was induced, and the peripheral vitreous was subsequently removed. With the aid of indocyanine green or brilliant blue dye, the epiretinal membrane and the ILM within 4PD were peeled. The inverted ILM flap technique with adjunctive autologous blood application was preferentially employed in some cases of MF and FD, particularly when OCT revealed significant attenuation in foveal thickness. This combined surgical approach was implemented to minimize the potential for postoperative macular hole development secondary to surgical manipulation. The choice of vitreous tamponade (filtered air, balanced salt solution, C3F8 gas, or silicone oil) was determined through comprehensive clinical evaluation, with selection criteria including: (1) duration of retinal detachment, (2) macular thickness measurements, and (3) individual patient compliance considerations.

### Statistical analyses

All statistical analyses were performed using IBM SPSS Statistics (version 24.0; IBM Corp., Armonk, NY, USA). The BCVA is expressed as the logarithm of the minimum angle of resolution (logMAR). The SE was calculated as the spherical dioptric power plus half of the cylindrical dioptric power. The normal distribution of each continuous variable was assessed with the use of the Shapiro–Wilk test. The differences in continuous variables between two groups were assessed using either the Mann–Whitney U test (for non-normally distributed continuous variable) or the two-sample *t*-test (for normally distributed continuous variable). Chi-square analyses were used to assess the relationships between categorical variables. Spearman correlation analysis was utilized to investigate the relationships between preoperative BCVA and the parameters obtained from OCT and OCTA, as well as the correlation between preoperative retinal sensitivity and the OCT and OCTA parameters. Univariate analyses of the associations between the prognostic factors for visual improvement were first performed. Significant factors were then used to construct multivariate linear and logistic regression models to assess the predictors of visual outcome. Statistical significance was set at *p* < 0.05.

## Results

This study included 23 eyes from 23 patients diagnosed with MF and FD. The participants had an average age of 51.38 ± 2.93 years, with six males (26.1%) and 17 females. The mean AL was 30.02 ± 0.31 mm, and the mean SE was −15.89 ± 1.02 diopters. Eighteen patients presented preoperative EZ disruption. Twelve phakic eyes underwent combined vitrectomy and cataract surgery, while eleven eyes were treated with only vitrectomy. Postoperative follow-up revealed no clinically significant cataract progression in the vitrectomy only group. Seventeen eyes received ILM peeling only, whereas six eyes performed ILM inversion along with autologous blood. Postoperatively, two eyes were filled with air, ten with C3F8, eight with silicone oil, and three with Balanced Salt Solution. Silicone oil tamponades were successfully removed during the follow-up period. All patients achieved complete resolution of foveal detachment, and none exhibit complication of macular hole or retinal detachment complications.

The logMAR BCVA improved from 0.46 ± 0.31 at baseline to 0.70 ± 0.42 (*p* < 0.001) at 6 months postoperative. Based on the BCVA assessed 6 months after surgery, patients with MF and FD were further categorized into two groups: those with BCVA ≥0.5 and those with BCVA <0.5. There were no significant differences between the two groups concerning age, gender, axial length, refraction, or retinal sensitivity (*p* = 0.259, 0.076, and 0.192, respectively; Table 1). However, the group with BCVA ≥0.5 exhibited a significantly lower FD height (333.56 ± 118.61 vs. 450 ± 105.49, *p* = 0.043) and a significantly higher VD in the SCP (43.34 ± 3.48 vs. 39.88 ± 2.07, *p* = 0.021) compared to the group with BCVA <0.5. No significant differences were found between the groups regarding preoperative BCVA, the area and perimeter of the EZ disruption, or DCP VD (*p* = 0.259, 0.076, and 0.192, respectively; see Table 1).

**Table 1:** Comparison of basic characteristics between the two groups separated by postoperative visual acuity.

Variables	Total	Postoperative BCVA ≥0.5 (*n* = 10)	Postoperative BCVA<0.5 (*n* = 13)	*p* value*
Demographics				
Patients/eyes, *n*	23	10/10	13/13	*-*
Sex (male/female), *n*	6/17	4/5	2/12	0.108
Age, years	51.38 ± 2.93	44.22 ± 11.41	54.08 ± 10.9	0.054
Axial length, mm	30.02 ± 0.31	29.8 ± 1.4	30.32 ± 1.44	0.438
SE, diopter	−15.89 ± 1.02	−13.97 ± 3.77	−17.09 ± 4.59	0.145
Baseline visual function				
Baseline BCVA, LogMAR	0.70 ± 0.09	0.52 ± 0.24	0.83 ± 0.49	0.088
2°MS, dB	12.51 ± 6.34	14.38 ± 5.57	10.42 ± 6.8	0.181
10° MS, dB	15.63 ± 6.04	17.18 ± 5.98	13.9 ± 5.95	0.248
20° MS, dB	17.24 ± 5.44	18.64 ± 4.86	15.68 ± 5.9	0.247
OCT parameters				
CFT, μm	588.74 ± 124.99	617.2 ± 122.84	566.85 ± 127	0.35
SCT, μm	52.39 ± 29.98	53.1 ± 35.73	51.85 ± 26.25	0.924
FD, μm	402.57 ± 128.51	328.9 ± 112.79	459.23 ± 112.84	**0.012**
FS, μm	640.22 ± 123.08	662.8 ± 102.62	622.85 ± 138.28	0.453
OCTA parameters				
Whole SCP VD, %	42.23 ± 3.32	43.76 ± 3.54	41.04 ± 2.71	**0.049**
ParaFovea SCP VD, %	20.03 ± 6.8	46.51 ± 3.57	44.13 ± 2.75	0.067
Fovea SCP VD, %	45.17 ± 3.29	22.98 ± 7.02	17.77 ± 5.91	0.085
Whole DCP VD, %	45.64 ± 5.11	45.16 ± 4.06	46 ± 5.93	0.705
ParaFovea DCP VD, %	32.86 ± 5.97	46.51 ± 4.42	47.98 ± 7.03	0.184
Fovea DCP VD, %	47.34 ± 5.96	35 ± 7.98	31.21 ± 3.31	0.570
FAZ area, μm^2^	0.26 ± 0.09	0.23 ± 0.1	0.28 ± 0.08	0.169
FAZ perimeter, μm	2.08 ± 0.42	1.92 ± 0.43	2.2 ± 0.39	0.104
Acircularity Index	1.17 ± 0.06	1.15 ± 0.05	1.19 ± 0.06	0.135
FD-300 area density, %	42.89 ± 4.9	44.93 ± 4.08	41.32 ± 5.03	0.079
FD-300 length density, %	12.47 ± 3.17	13.72 ± 3.32	11.5 ± 2.81	0.097
En-face OCT parameters				
EZ disruption Area, μm^2^	1.18 ± 1.33	0.96 ± 1.49	1.4 ± 1.19	0.496
EZ disruption Perimeter, μm	4.68 ± 3.39	3.67 ± 3.57	5.69 ± 3.07	0.217

We analyzed the relationship between preoperative visual function and structural parameters in patients with MF and FD. The results indicated that preoperative BCVA was significantly correlated with CFT (*r* = −0.53, *p* = 0.01), the area of EZ disruption (*r* = 0.63, *p* = 0.01), and the perimeter of EZ disruption (*r* = 0.68, *p* = 0.01). Additionally, the preoperative 20° MS was significantly correlated with SCT (*r* = −0.56, *p* = 0.01), whole DCP VD (*r* = 0.65, *p* < 0.001), parafoveal VD (*r* = 0.60, *p* = 0.01), as well as the area of EZ disruption (*r* = −0.68, *p* = 0.01), and the perimeter of EZ disruption (*r* = −0.60, *p* = 0.02). The preoperative 10° MS was significantly correlated with SCT (*r* = −0.54, *p* = 0.02), whole DCP VD (*r* = 0.58, *p* = 0.01), parafoveal VD (*r* = 0.51, *p* = 0.03), as well as the area of EZ disruption (*r* = −0.70, *p* = 0.01), and the perimeter of EZ disruption (*r* = −0.66, *p* = 0.01). The preoperative 2° MS was also significantly correlated with SCT (*r* = −0.62, *p* < 0.001), whole DCP VD (*r* = 0.53, *p* = 0.02), parafoveal VD (*r* = 0.46, *p* = 0.05), as well as the area of EZ disruption (*r* = −0.55, *p* = 0.04), and the perimeter of EZ disruption (*r* = −0.59, *p* = 0.03) (see Tables 2, 3).

**Table 2:** Correlation between preoperative OCTA, OCT variables, and visual function in patients with MF and FD.

Variables	LogBCVA (*n* = 23)		20° MS (*n* = 19)		10°MS (*n* = 19)		2°MS (*n* = 19)	
	*r*	*p*	*r*	*p*	*r*	*p*	*r*	*p*
Demographics								
Age, years	0.28	0.21	−0.37	0.13	−0.47	0.05	−0.42	0.08
Axial length, mm	0.09	0.69	−0.24	0.38	−0.16	0.55	−0.31	0.25
SE, diopter	−0.27	0.29	−0.07	0.83	−0.16	0.58	−0.14	0.64
OCT parameters								
CFT, μm	**−0.53**	**0.01**	−0.25	0.31	−0.24	0.32	−0.27	0.27
SCT, μm	0.02	0.93	**−0.56**	**0.01**	**−0.54**	**0.02**	**−0.62**	**0.00**
FD, μm	0.06	0.77	0.15	0.55	0.10	0.69	0.10	0.69
FS, μm	−0.29	0.18	−0.37	0.12	0.45	0.45	−0.20	0.41
OCTA parameters								
Whole SCP VD, %	0.18	0.42	0.02	0.94	0.12	0.63	0.25	0.30
ParaFovea SCP VD, %	0.10	0.66	0.01	0.96	0.07	0.77	0.22	0.36
Fovea SCP VD, %	0.34	0.12	−0.06	0.82	0.12	0.64	0.14	0.58
Whole DCP VD, %	0.04	0.87	**0.65**	**0.00**	**0.58**	**0.01**	**0.53**	**0.02**
ParaFovea DCP VD, %	−0.02	0.92	**0.60**	**0.01**	**0.51**	**0.03**	**0.46**	**0.05**
Fovea DCP VD, %	0.41	0.06	0.12	0.64	0.29	0.24	0.30	0.22
FAZ area, μm^2^	−0.33	0.13	0.12	0.61	−0.08	0.75	−0.03	0.89
FAZ perimeter, μm	−0.29	0.17	0.17	0.49	−0.03	0.89	0.00	1.00
Acircularity Index	−0.11	0.63	0.06	0.81	−0.04	0.87	−0.06	0.80
FD-300 area density, %	0.34	0.11	0.13	0.60	0.17	0.49	0.32	0.18
En-face OCT parameters								
EZ disruption Area, μm^2^	**0.63**	**0.01**	**−0.68**	**0.01**	**−0.70**	**0.01**	**−0.55**	**0.04**
EZ disruption Perimeter, μm	**0.68**	**0.00**	**−0.60**	**0.02**	**−0.66**	**0.01**	**−0.59**	**0.03**

Bold values represent statistically significant results.

**Table 3:** Univariate and multivariate regression analysis of the factors associated with postoperative visual acuity.

Variables	Univariate linear regression analysis			Multivariable linear regression analysis		
	Unstandardized Coefficient *β*	Standardized Coefficient *β*	*p*	Unstandardized Coefficient *β*	Standardized Coefficient *β*	*p*
Demographics						
Sex	−0.223	−0.338	0.115			
Age, years	0.009	0.357	0.094			
Axial length, mm	0.035	0.169	0.476			
SE, diopter	−0.032	−0.442	0.075			
Baseline visual function						
Baseline BCVA, LogMAR	**0.494**	**0.674**	**<0.001**	**0.425**	**0.646**	**0.012**
2°MS, dB	−0.012	−0.286	0.234			
10° MS, dB	−0.012	−0.291	0.227			
20° MS, dB	−0.013	−0.265	0.274			
OCT parameters						
CFT, μm	−0.001	0.403	0.056			
SCT, μm	0.000	0.202	0.356			
FD, μm	−0.000	−0.042	0.849			
FS, μm	−0.001	−0.249	0.251			
OCTA parameters						
Whole SCP VD, %	**−0.042**	**−0.452**	**0.030**	**−0.044**	**−0.469**	**0.025**
ParaFovea SCP VD, %	−0.017	−0.410	0.052			
Fovea SCP VD, %	−0.039	−0.375	0.078			
Whole DCP VD, %	0.013	0.221	0.311			
ParaFovea DCP VD, %	0.013	0.257	0.236			
Fovea DCP VD, %	−0.016	−0.300	0.164			
FAZ area, μm^2^	1.333	0.379	0.075			
FAZ perimeter, μm	0.278	0.376	0.077			
Acircularity Index	1.015	0.195	0.373			
FD-300 area density, %	−0.021	−0.328	0.127			
FD-300 Length Density, %	−0.039	−0.400	0.059			
En-face OCT parameters						
EZ disruption Area, μm^2^	0.071	0.306	0.216			
EZ disruption Perimeter, μm	**0.047**	**0.514**	**0.029**	**−0.013**	**−0.147**	**0.560**

Bold values represent statistically significant results.

In the univariate regression model, postoperative BCVA at 6 months was significantly correlated baseline BCVA (*β* = 0.674, *p* < 0.001), EZ fracture perimeter (*β* = 0.514, *p* = 0.029) and whole SCP VD (*β* = −0.452, *p* = 0.030). The multivariable analysis included variables that showed a *p* value of less than 0.05 in the univariate analysis. The results indicated that only preoperative BCVA (*β* = 0.646, *p* = 0.012) and whole SCP VD (*β* = −0.469, *p* = 0.025) were substantially correlated with postoperative BCVA at 6 months.

## Discussion

To the best of our knowledge, this study represents the first attempt to use OCTA flow and structural parameters to predict visual prognosis in patients with MF and FD following vitrectomy. Our findings indicate that improved preoperative BCVA and higher whole SCP VD are significantly associated with better visual outcomes.

The splitting of interlayer structures in the retina of patients with MF poses significant challenges for layer segmentation, which explains the limited literature utilizing OCTA to investigate flow and structural parameters in these patients. In this study, we manually corrected segmentation errors in the OCTA images of patients with MF and FD, achieving clear visualization of both the superficial and deep capillary flow images of the retina. However, in patients with inner layer splitting, it is difficult to differentiate between the capillaries of the nerve fiber layer and those of the ganglion cell layer due to the presence of columnar structures extending from the INL to the ILM. Consequently, our analysis relied on the established traditional vascular networks of the SCP and DCP, each comprising two vascular complexes.

It is reported to be challenging to assess the degree of EZ damage in eyes with MF and FD (17). However, when using the en-face images of outer layer acquired from OCTA, we observed that regions of EZ fracture appeared brighter due to increased light transmission in the absence of the EZ (19–21). Thus, we can quantitatively measure the area and perimeter of the EZ fracture using these en-face images.

Although many studies have reported structural and functional changes associated with myopia, there is limited research focusing on the correlation between structural and functional changes in patients with MF. Our study indicates a significant correlation between preoperative BCVA and CFT in patients with MF and FD, which is consistent with previous findings (22). Additionally, we found a significant correlation between BCVA and the area and perimeter of the EZ disruption. Previous studies (23, 24) have reported significant relationship between the integrity of the EZ line and visual function in various retinal diseases. This aligns with our clinical observations that patients with larger schisis and more extensive EZ disruption tended to exhibit poorer visual acuity. The visual function findings concerning MS are interesting, we revealed significant correlations between MS at 20°, 10°, 2° with SCT, DCP VD, and both the area and perimeter of the EZ disruption. However, the relationship between MS and CFT is not as evident. Park et al. (24) has reported that MS in high myopia is reduced in patchy atrophic lesions and related to the integrity of the RPE, EZ, and external limiting membrane (ELM), but not associated with outer retina schisis, which somewhat supports our findings. Therefore, we can conclude that in patients with MF and FD the area and perimeter of the EZ disruption can impact both visual acuity and retinal sensitivity, while the extent of the schisis mainly affects visual acuity, the deep retinal blood flow and choroidal supply mainly relate to retinal sensitivity. Therefore, in addition to visual acuity, retinal sensitivity may serve as another important indicator for monitoring changes in visual function in MF patients.

Lehmann et al. (25) categorized 65 patients with MF into four groups based on their preoperative visual acuity, demonstrating that preoperative BCVA was the only independent predictor of final BCVA. Fujimoto et al. (26) examined 17 eyes with MF and found that the final integrity of the EZ and ELM layers was significantly associated with final BCVA, while preoperative SCT showed no correlation with postoperative BCVA. Lim et al. (16) found in their multivariable analysis that patients with MF who have preoperative EZ disruption often have poorer visual outcome. Lee et al. (27) discovered that good preoperative visual acuity and the absence of FD before surgery are important predictors of good visual prognosis, while CFT was not significantly correlated with postoperative BCVA. Sborgia et al. (14) found that final BCVA was correlated with preoperative BCVA, postoperative CFT, and the recovery status of the EZ at six months. Iida et al. (28) examined 11 eyes with MF, among which 8 eyes exhibited FD. Their findings revealed that the postoperative BCVA 12 months after surgery had a significant correlation with age, preoperative BCVA, preoperative SCT, and posterior staphyloma height. However, the authors did not perform a multivariable linear regression analysis to identify independent factors related to postoperative BCVA.

Consistent with above studies, our research demonstrated that good preoperative visual acuity correlates with a favorable visual prognosis. Furthermore, our research is the first to identify that preoperative whole SCP VD is an independent predictor of postoperative BCVA 6 months after surgery. Patients with postoperative BCVA ≥ 0.5 exhibited significantly higher SCP VD compared to those with BCVA < 0.5. Although MF and FD primarily involve pathologies in the outer retina, our study only detected a correlation between postoperative BCVA and SCP VD, with no significant association observed with DCP VD. We hypothesize several potential explanations for these findings. First, although our study employed projection artifact removal technology, residual projection artifacts may still persist in some patients, especially in eyes with a particularly thinned retina. Current OCTA technology does not yet permit their complete eradication, potentially leading to inaccurate DCP VD measurements in certain patients with MF and FD, thereby obscuring correlation between DCP VD and postoperative visual acuity. Second, we propose a pathophysiological hypothesis that in MFand FD patients demonstrating concurrent choroidal vascular insufficiency and impaired SCP perfusion, compensatory hemodynamic adaptations may occur in DCP. These speculations finds partial support in our dataset, where we observed numerically higher (though statistically non-significant) DCP VD values in the postoperative BCVA <0.5 group compared to the BCVA ≥0.5 group. Further studies with larger scale sample size will be necessary to confirm the precision and generalizability of our findings and to elucidate the exact pathophysiological mechanisms underlying these observations.

Our study had several limitations that should be acknowledged. First, it was a retrospective study with relatively small sample size, further studies with larger sample sizes are warranted to validate our observations and explore the underlying mechanisms. Second, the follow-up period in our study was relatively short. Longer follow-up would be beneficial to assess the durability of the observed effects and to identify any late complications or changes in visual function that may arise over time. Third, although this study included patients who underwent different surgical techniques, we focused on preoperative characteristics on postoperative visual prognosis rather than the effect of the surgical technique.

In conclusion, our findings indicate that among the various preoperative factors, good visual acuity and higher SCP VD before surgery are predictors for favorable visual outcomes in patients with MF and FD following vitrectomy. Our study highlights that OCTA could potentially assist in clinical decision-making and patient management for MF and FD cases.

## Data Availability

The raw data supporting the conclusions of this article will be made available by the authors, without undue reservation.
